# Harmonizing patient-centric requirements for secure digital health services in heterogeneous settings

**DOI:** 10.1186/s12913-024-11978-x

**Published:** 2025-02-11

**Authors:** Luigi Assom, Thashmee Karunaratne, Aron Larsson

**Affiliations:** 1 Department of Computer and Systems Sciences, Stockholm University, Kista, 164 25 Sweden; 2 School of Industrial Engineering and Management, Royal Institute of Technology/KTH, Stockholm, 100 44 Sweden; 3 Forum for Digitalisation, Mid Sweden University, Sundsvall, 851 70 Sweden

**Keywords:** Patient-centric care, Under-served areas, Harmonization requirements, Electronic journal systems, Knowledge graphs

## Abstract

Implementing electronic health services with a patient-centric focus while adapting to the know-how of local contexts is a challenge. This paper addresses this challenge by establishing a template of modular requirements for designing a viable Electronic Health Record (EHR) system that enables transmission and sharing of patient data across primary, secondary, and specialized care, ensuring versatility in diverse healthcare environments and across varying socio-economic landscapes. The research is anchored in design science and employs an action research strategy, using northern Brazil as empirical case. The approach builds on generic requirements from standards established by the European Union, Fast Healthcare Interoperability Resources (FHIR), and the Swedish ePrescription journal system. These requirements are refined and adapted to the Brazilian context through a participatory method, considering development disparities across municipalities and aligning with national policy. A key feature is the integration of knowledge graphs, which, when combined with fieldwork iterations involving healthcare professionals and patient association representatives, facilitated the extraction of patient-centric requirements. Strategies from Brazilian healthcare policies targeting chronic kidney disease, selected as a significant challenge for specialized healthcare in emergent areas, were incorporated to generalize the design of EHR modules aimed at prevention and monitoring of population at risk. Results support that harmonization towards legacy system is strongly advised and discourage the introduction of systems designed from scratch.

## Introduction

Universal health coverage (UHC) has a causal effect in reducing societal inequality and poverty [1]. In contrast, poverty restricts access to quality health care and healthier lifestyles. Relative poverty (income below 60% of the median) affects health outcomes and increases government healthcare costs [2, 3]; for example, 25% of primary and acute care spending in the UK is attributed to treating people in poverty [4].

A global survey of World Health Organization (WHO) highlights that a holistic view over processes, structures, roles, standards, and legislation is at least as import as technological investments for systemic transformation of healthcare through eHealth services [1]. In 2015, 40% of EU countries lacked national electronic health records (EHR) systems, and half had no patient-access legislation, with funding cited as the main barrier [1]. It is assumed that the WHO’s recommendations, although EU-specific, are relevant for guiding digital health in other Countries with similar challenges and in particular to under-served areas, since inadequate digital systems, legislation, and funding disproportionately impact those regions.

The integration of health services across medical, public health, social care and prevention is considered a necessary action to design health systems that are capable of tackling the complexity of interplaying factors causing poverty [2], therefore expected to enhance the handling of patients. Digitization of health care, i.e. eHealth, is expected to provide an infrastructure to improve the efficiency of such integrated services and also reduce costs by mitigating barriers to access, thus pushing forward the agenda to minimize health disparities exacerbated by relative poverty. With this lens, eHealth is considered a premise for improving the delivery of healthcare services towards the most vulnerable cohorts of patients living in low-resource or under-served areas. eHealth is therefore considered a strategic asset for implementing a portfolio of public and private health services, which necessitate coordinated planning, implementation, and evaluation of patient-centric services across various and heterogeneous regions. Two main challenges concern the harmonization between different administrative regions and administrative scales, and patient centricity: harmonization is a subset of interoperability necessary to ensure continuity of care and efficient management of patients data [5]; patient-centricity concerns prioritizing the needs, preferences, and experiences of end-users, under the constraint of exchanging data secured by electronic identification (i.e. user centricity) [6]. With this regards, harmonization aims to lead to an acceptance of new processes and working patterns by stakeholders affected by the introduction of new information systems, while acknowledging different working contexts and capacity to conform to standards [7].

The study, set within an international cooperation project between Sweden and Brazil, explores innovative solutions to support the implementation of an electronic health record (EHR) journal system through a patient-centric approach. Given that the study inherently involves a problem-solving approach and the relevance of digital health transformation in the scope of universal health coverage, we referred to design science as theoretical ground and defined the problem space through: *a)* changes imposed by Brazil’s Digital Health strategies (environmental context), and *b)* demand of electronic identity (eID) in healthcare (opportunity-problem) [6, 8].

Our main contribution is a set of minimum requirements for a patient-centric electronic journal system to enable transmission and access of patient histories throughout referral and counter-referral processes. We combined knowledge graphs and field data to generate requirements compliant with FHIR and EU principles, then harmonized with the Brazilian health-care system.

## Background

Harmonization is considered a digital premise to enable patient-centric services, being “the minimum denominator for interoperability to exist” that should “include a common language and shared understanding of the image of information systems” [9]. Beyond a technical challenge, harmonization and patient-centricity are a strategic imperative to enhance quality and efficient delivery of healthcare services in in many national policies, especially for under-served regions relying on paper-based data transmission, which causes issues like incomplete patient records, mismatched medication stocks, and data duplication (e.g. omissions of allergies to medications; risks of disrupted care during life-long therapies, and erroneous handling of homonyms).

Digital transformation has a dual impact on organizations: it can threaten existing practices by requiring a redefinition of services and staff roles, or create opportunities to enhance operations by empowering staff with the necessary training and resources to adapt to new technologies [10]. This dynamic is relevant and sensitive in healthcare, where integrating digital tools can significantly alter both clinical and administrative processes. For example, a hospital’s digital transformation aimed at improving efficiency and patient-centricity was found to affect administrative patient management rather than clinical workflows or roles of healthcare professionals (HCPs) [10]. The authors recommended digital transformation efforts to be focused on understanding “‘what’ work is transformed” and which socio-technical barriers are faced by actors, rather than simply analyzing policy impacts on management and staff (e.g. impositions of new procedures and conflict-reconciliations processes).

### Harmonization and patient-centricity in eHealth – European strategy

The European Union (EU) has made significant strides in this regard, with guidelines for the adoption of standards and of systems compliant with the electronic identification (eID) [11, 12], which is considered a requirement to simplify processes such electronic Prescriptions (ePrescriptions) [13] and transmission of patient data in cross-border use cases [14].

The European Digital Identity Wallet (EUDI Wallet) [15] is designed to ensure interoperability through administrative scales and differences (i.e. across national- and cross-borders of EU member states) by allowing an electronically secured access and verification of identities and attributes: in the health context, simplify access to ePrescription and to patients anamnesis (p. 11 in [15]), providing options for on-boarding processes that require mandatory documentation and medical certificates authorizing medical procedures [6].

Specifically, the EUDI wallet supports both qualified and non-qualified electronic attestations of attributes, which are necessary for the harmonized implementation across healthcare domains as described in EU regulation “Electronic Identification And Trust Services” (eIDAS). The eHealth Network (a voluntary network set up under article 14 of Directive 2011/24/EU providing technical support to competent authorities dealing with eHealth) proposed examples of ePrescription and eDispensations use cases, where users can access their prescriptions and anamnesis data at any time, at any pharmacy or doctor operating in any municipality of any EU member state [13].

### Successful implementations of ePrescription systems – The case of Sweden

Computerized provider or physician order entry (CPOE) enabling HCPs to electronically order drugs and tests have been in development since the 1970s, first implemented in hospital usage at Wishard Memorial Hospital in 1984 [16]. However, a more widespread utilization of digital technology for managing prescriptions, allowing for HCPs to exchange patient data with pharmacies and social insurance systems and the prescription process, took rise in the 2010s, with countries such as Denmark, The Netherlands, Estonia, and Sweden as early front-runners [17].

The WHO report analyzed the implementation of the ePrescription in Sweden as one of the most advanced success case studies of eHealth [1]. The initial pilot tests for digitizing the Swedish health journal system started with patient-held smart cards in 1987. The scope and security of ePrescription were enhanced by the adoption of EDIFACT^1^ syntax during the 1990s, later evolved in an XML-based standard for management of pharmaceuticals to diagnosis. This standard was subject to be further improved by the introduction of object-oriented modeling, matching the recommendations of the international standard for interoperability and transmission of health-care data “Fast Healthcare Interoperability Resources” (FHIR) [18, 19]. By 2012, Sweden achieved a complete transition to an electronic national EHR system (a 100% coverage of EHR system), with a significant 90% issuance rate for electronic prescriptions to date providing comprehensive access to medication histories of patients [18].

With respect to user-centricity, physicians’ attitudes towards the Swedish EHRs revealed broad acceptance early. In a 2009 survey [20] electronic prescriptions was concluded to be deemed safer than handwritten ones. Some formative qualitative recommendations were addressed to mitigate weak points of other EHR implementations, such as reducing the complexity of interfaces (noted as “too many mouse clicks”), a need of drug-selection functions including the need of issuing receipt transmission of prescriptions and of establishing routines for handling disruptions allowing asynchronous prescription transfers during system down-times, mandatory requirements for prescription checks by the professionals before sending to pharmacies, and clear display of patient names and their social security numbers. Since then, the Swedish case of eHealth penetration and digital health services has been evaluated in different benchmarks since 2016 and the results have been congruent between these assessments in that the eHealth services provided have persisted, resting upon actionable national strategies [21] while also experiencing positive acceptance from citizens [22] and satisfying levels of usability over time [23, 24]. The Swedish, as well as the Nordic, ePrescription system has further been well scrutinized in literature and is subject to multiple studies, enabling for a good understanding of problems and issues that remain and their sources [25].

The two emphasized benefits of the Swedish ePrescription system are the delivery of medication by qualified professionals even without printout prescriptions, and the possibility to verify prescriptions compatible with the patients’ historical records [1]. Such features are judged to be of value for the Brazilian case, as storing and exchanging clinical information longitudinally, harmonized with information systems for “laboratory, billing, pharmacy or inventory management”, are emphasized requirements for customizable telemedicine platforms in under-served areas [26].

### Harmonization and patient-centricity in eHealth – Brazilian strategy

Brazil’s Digital Health Strategy prioritizes analyzing “both national and international experiences with personal health record systems“ to ensure interoperability and adherence to best practices (see p. 66 in [27]), which is necessary for maintaining continuity of care. Harmonization efforts should initially focus on primary health care systems (see pp. 29, 32 in [28]).

The strategy emphasizes leveraging both “national and international experiences in the use of personal health record systems to ensure interoperability with other systems”, positioning harmonization as key to enabling the National Healthcare Data Network (RDNS) to deliver essential digital health services (see p. 66 in [27]). This involves adopting standards that support integration and widespread use across the Country.

The aim is to ensure continuity of care by harmonizing primary healthcare systems (see: pp. 29; 32 in [28]). Key governmental programs include the “Conecte SUS Program”^2^, “Informatiza APS Program”^3^, and the “Pilot Project Supporting the Informatization of Primary Healthcare”^4^. Pilot projects integrating RDNS were tested in ten cities across across various states^5^ from 2019 to 2020, at the beginning of an eight-year strategy (see: pp. 43; 61 in [28]). Users should have “access [EHR] management without regional access restrictions”, enable integration of “multiple organizations [..] for scheduling appointments and exams” and the “knowledge extraction for improving diagnosis and evaluation of therapies” (see p. 77 in [27]). The design process should involve “SUS stakeholders and managers [..] to identify priorities, roles, responsibilities, expected results and goals for population health” for implementing telehealth services that can be “integrated with [existing] healthcare [..] processes” (see p. 58 in [27]).

Brazil’s policy also focuses on prevention and monitoring of chronic kidney diseases as actions to address UHC in emerging areas [29]. Monitoring prevention campaign effectiveness and third-party services contracted in specialized care (e.g. private structures; transportation services and domestic assistance for periodic patient care) are considered essential [30]. A modular approach to integrating these functions into EHR systems, emphasizing harmonization and patient-centricity, is expected to support UHC cost-effectively, even with minimal capital investments for adding new extentions [26].

Harmonization is especially critical for care continuity in remote areas. It can address challenges such as optimizing appointment scheduling to reduce long-distance travel, managing financial assistance for low-income patients, and tracking public funds negotiated with private care [30, 31], enabling comprehensive evaluations of programmatic and clinical outcomes in under-served areas [26].

## Research approach

Even though electronic identification (e-ID) journals are mature technologies with established descriptive knowledge, their application in under-served areas presents unique challenges that justify the use of Design Science Research (DSR), as it enables the creation and rigorous assessment for extending known technical solutions to new problems [32].

The novelty of the context arises from the distinct socio-economic challenges and infrastructure gaps in Brazilian municipalities, with administrations marked by high differences in access to resources, which require the adaptation and re-evaluation of technical solutions as eIDAS and EHR systems.

Action research was adopted as the strategy for planning longitudinal fieldwork iterations. This approach was used to define processes, co-create requirements, and understand how healthcare actors currently operate and interact within the health systems. The knowledge contribution was framed as an exaptation process, extracting design principles from eID-enabled healthcare journals in Europe and Sweden and adapting them for patient-centricity in the Brazilian context.

Action research was also employed as a method to investigate and co-create solutions to emerging problems. The intervention targeted audiences of HCPs and patient representatives to scope digitalization in terms of acceptability (i.e. the capacity to sustain current clinical procedures), implementation (e.g. fidelity to support correct practices) and adaptation (i.e. the capacity to support under-served municipalities) [33]. The methodology evolved from designing general diagrams, representing high-level processes in primary, secondary, and specialized care (using the software “Miro”^6^), to creating knowledge graphs with prescriptive requirements for implementing FHIR-compliant modular EHR systems. The description of the knowledge graphs can be also executed to instantiate a graph data-base prototype.

Specifically, we aimed to adapt and refine requirements for handling patients between primary, secondary and specialized healthcare, with a special focus on municipalities with limited resources, and to map processes that are robust to different administrative complexities (i.e. high discrepancy of resources and capacity to attend to patients depending on healthcare units and geographical context) and patient needs (e.g. patients living in remote or under-served areas).

A significant aspect of our research involved decomposing the issue of manual patient record handling into manageable units, identified initially as selected use cases in primary, secondary and specialized health care. Specialized care was represented by chronic kidney disease, as an example of a condition that severely plagues remote areas and requires special attention from Brazilian policies aimed at effective prevention campaigns [30, 34, 35]). Fieldwork iterations were planned to comprehend the fundamental problems affecting patient-centricity in the selected use cases and then select a common denominator of issues affecting all the use cases, which was identified in the practice of manual transmission of Patient records impeding the access to exhaustive patients’ anamnesis and history records through referral and counter referral practices.

The scope of the fieldwork was limited to validate the mapping of processes and requirements from 26 municipalities from the State of Maranhão. The majority of the municipalities involved were operating in emerging areas, and there was at least one representative for each administrative level of Brazilian health care (municipality-, regional-, state-level). Hence, we considered the sample of respondents adequate for eliciting sets of requirements for patient-centricity in under-served areas that are satisfactory under the constraints of selected use cases that were highlighted by HCPs in the first fieldwork iterations and then validated and refined by Patient Associations. The observations of complex processes through meetings, interactions and webinars allowed us to analyze in-depth the processes through which actors address patient-centricity in challenging situations (e.g. municipalities with lack of resources or in remote areas).

We considered this sample appropriate to tackle a “‘grand challenge’ [through an inductive method] requiring extensive collaboration and coordination among actors and technologies” [36], such as requirement elicitation for continuous health care in under-served areas, enhancing interoperability with areas of greater capacity. We also considered them functional to mitigate the risk of innovation-related barriers, “mostly related to the process stage development and design”, and of contextual barriers such as different geopolitical contexts and policies, for developing digital artifacts requiring electronic identification [37], in perspective of extending this study towards implementation of a Minimum Viable Product (MVP) of EHR with potential replication in other regions. Hence, this study aims to address the significant knowledge gap in eliciting a set of requirements for an MVP EHR to enhance continuity of care in emergent areas with a patient-centric approach. The grounding requirements for patient centricity, privacy and security of patient data are drawn from EU policies and the Swedish journal system. Requirements for harmonization are drawn from EU and Brazilian policies: the first, as an example of harmonization for national and international interoperability through EU member countries, to be adapted to the heterogeneous complexity of Brazilian health care.

### Method

The exploratory question to infer sets of satisfactory requirements for EHRs justifies action research methods, with a focus on qualitative and cross-contextual analysis. We planned for action research instead of a case study, because we considered that the “original intent [of the authors] is to research while effecting change” and being participants rather than “independent observers” [38], since this research was conducted in the realm of bilateral cooperation between Sweden and Brazil. Within this context, qualitative research is scoped for patient centricity and for part of harmonization requirements concerning the IT infrastructures currently in use in Brazilian primary and secondary care units.

Qualitative research contextualized the formative requirements to scope the compliance of digital health intervention with electronic identification and secured transmission of patient data within clinical practices, drew from the WHO’s assessment of the European context, the Swedish Journal system and best practices for maintaining FHIR-compliant systems. With this regard, legislation requirements and guidelines in Europe are taken into account as a framework for structuring journal systems on top of electronic identification with an automated Certificate Life-cycle Management [15, 39–44], required to be compliant with the eIDAS Regulation. The technical guidelines for electronic identification and eHealth taxonomies were based on design principles in the EU [45, 46].

System integration requirements specific to interface electronic identification and patient data with the Brazilian primary care infrastructure were drawn from Brazilian policies [27, 28, 47].

**Fig. 1:**
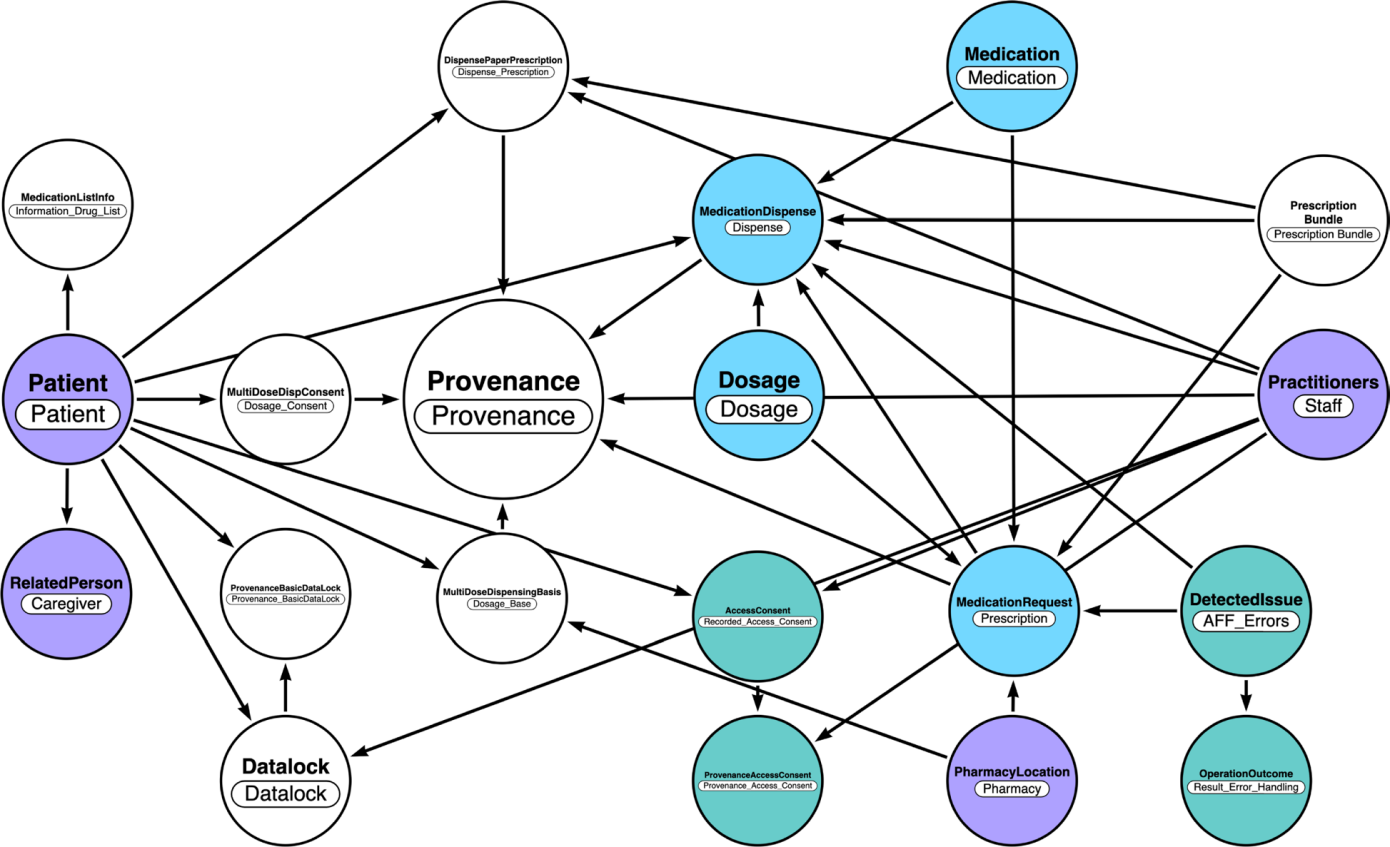
Information specification describing the use of FHIR resources in the Swedish National Medicines List. Legend - *Purple:* resources reflecting Patient and Practitioners; *Teal:* resources reflecting Device compartment; *Azure:* modules describing medication prescription; White: module Provenance and associated resources utilized for assessing authenticity, enabling trust, and allowing for reproduction

The Swedish system is taken as a European reference for implementing ePrescription and medication journals, [48, 49] to infer best practices for implementing FHIR-compliant journals. FHIR documentation prescribes a modular implementation of systems, such as templates of data objects that can be flexibly extended with eHealth taxonomies for designing application interfaces (RESTful APIs) or knowledge graphs [19]. As example, Fig. 1 shows a knowledge graph utilizing the FHIR taxonomies utilized by the Swedish E-Health agency for ePrescription of medications (see: Table 1 and supplemental material in the digital repository).

**Table 1:** Information specification of FHIR resources in the Swedish National Medicines List

Entities (FHIR 5.0 resources)	Use case (description)
MedicationRequest	Prescription-related functionalities
Patient	Central to patient-related information
Practitioner	Healthcare provider information
Dosage	Detailing medication dosages
MedicationDispense	Handling medication dispensing events
DispensePaperPrescription	Paper prescription dispensing
Provenance	For tracking the origin of data
Datalock	Data access control
MultiDoseDispConsent	Multi-dose dispensing consent
RelatedPerson	For information about related persons like guardians
MultiDoseDispensingBasis	Basis for multi-dose dispensing
MedicationListInfo	Medication list information
PharmacyLocation	Information about pharmacy locations
DetectedIssue	For identifying and addressing issues in healthcare data
OperationOutcome	Handling results and errors in operations
Medication	Information about specific medications
PrescriptionBundle	Handling multiple prescription-related resources
Dispatch	Handling multiple prescription-related resources
ConceptMap	Mapping concepts between different systems
ValueSet	Defining sets of codes and values for specific purpose
AccessConsent	Managing consent for accessing healthcare data
Provenance	Managing consent for accessing healthcare data
MedicationRequest	Prescription-related functionalities

One advantage of opting for knowledge graph representations over RESTful APIs is the simplified maintenance achieved by mapping healthcare processes to the data structures specified by FHIR. This approach provides frameworks that simultaneously represent both the semantic and data structure aspects of healthcare processes [50–52].

As an example of a significant specialized care in under-served areas, chronic kidney disease is selected due to the associated economic burden on behalf of patients (e.g. prohibitive time travel and distance can affect continuity to attend for dialysis; “treatment [costs] of milder forms of chronic kidney disease [appearing] much greater than end-stage of kidney disease”) and lack of access to drinkable or sanitized water, which characterizes many of the emergent areas in the World [34]. Requirements for addressing chronic kidney diseases were extracted from the point of view of prevention and continuous care of vulnerable population cohorts living in remote areas [30]. Handling of specialized care was drawn from legislation and guidelines focused on this disease and on remote areas constraints, on purpose to design modular extensions of EHR that are harmonized with referral and counter-referrals with primary and secondary care and can integrate functions to trace the impact of prevention and monitoring of chronic diseases in emerging municipalities [5, 27, 28, 30, 53–55]).

The general requirements lists were finally adapted to emerging areas in Brazil through iterative fieldwork research and data collection.

### Data collection

Data collection was planned to validate and converge data gathering in three steps.

**Fig. 2:**
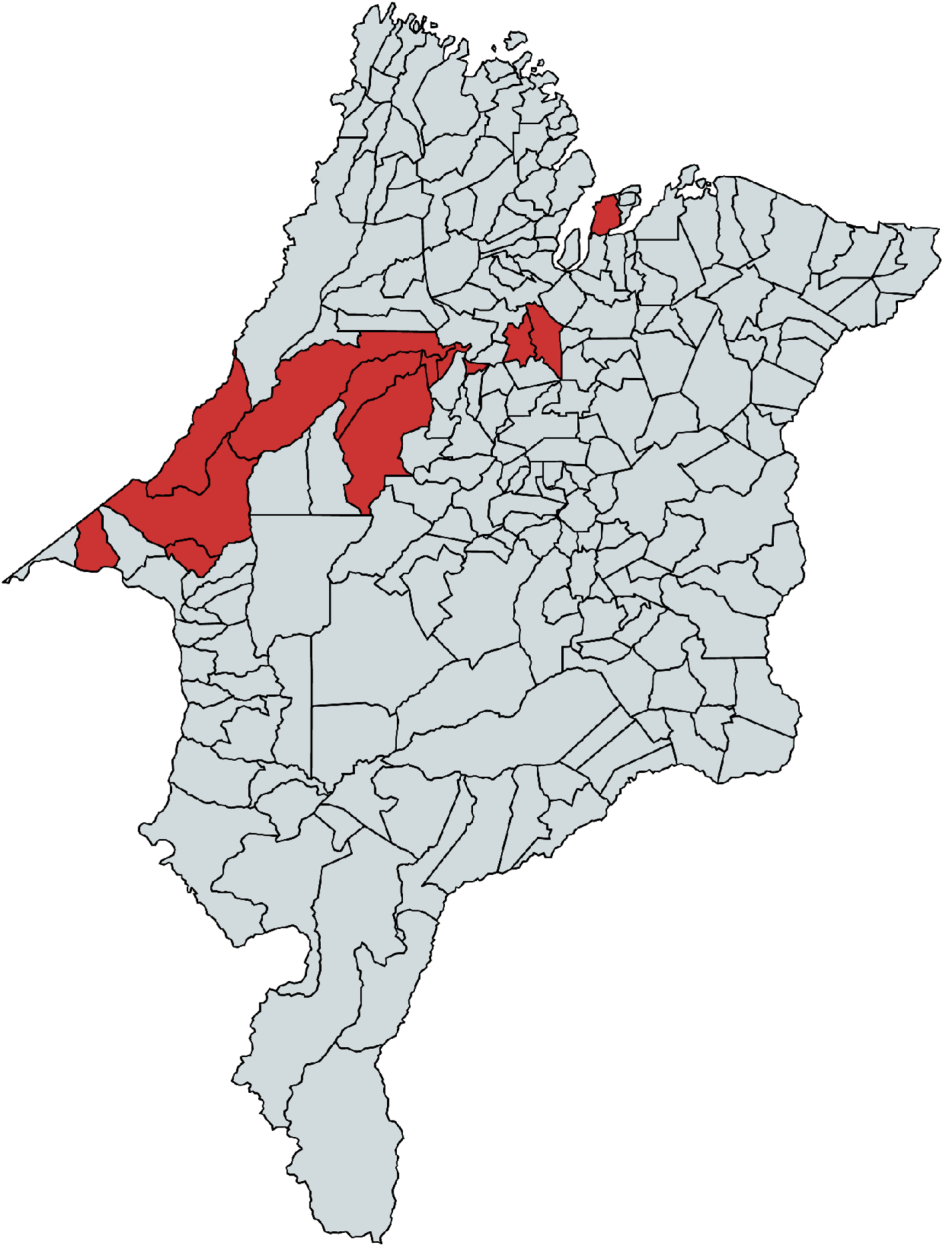
Selected municipalities from the State of Maranhão (Brazil): São Luís, São Francisco de Brejão, Itinga do Maranhão, Vila Nova dos Martírios, Santa Luzia, Açailândia, Vitória do Mearim, Bom Jardim, Alto Alegre do Pindaré, Bela Vista do Maranhão, Tufilândia, Alto Alegre do Pindare’, Vila nova dos San Martirios Sao Francisco de Brejao

The first step administered on-site workshops and a questionnaire to HCPs to identify common and specific problems affecting primary and secondary health care (November 2023). Participants were HCPs representatives from municipalities from the State of Maranhão (see: Fig. 2), primarily IT professionals and HCPs classified as *primary stakeholders* of EHRs according to [56], as they have direct access to patient data.

Workshops included discussions to co-create a preliminary Miro board illustrating high-level diagrams of health-care, which led to define nine use cases: *Consultation*; *Prescription*; *Diagnostic Treatments procedures*; *Referrals* to primary or secondary care; *Counter-Referrals* between secondary and primary care; *Execution of Treatments plans*; *Parental Care*; *Elderly Health controls*; *System Administration procedures* for reporting and management of patient-data. The questionnaire, developed specifically for this study, comprised a set of five open-ended questions to elicit, for each use case and for each municipality: the most problematic issues faced by Patients and by HCPs; examples of criticality; administrative responsibility and digital tools used in the municipality to handle patients records and follow-ups. Answers were analyzed through document and thematic analysis to produce, for each use case, the following lists: identified themes and the corresponding problems common to all municipalities; elicited general requirements to address common problems; and specific requirements for selected municipalities (usually focused on administrative needs, like enhancing capacity with specific digital tools or IT services). Collected results were used as data for the second step. The questionnaire, anonymous answers and results are available as supplemented material (see: “Anonymized Use Cases Workshops” and “Analysis - Workshops - Cases” in the digital repository at: Data availability).

The second step administered on-site workshops to validate extracted problems and to prioritize the use cases (March 2024). A sample of 3 to 10 participants represented municipalities at local, regional and state levels in the State of Maranhão.

*Referral* and *Counter-referral* were prioritized because it was found they underlie and critically affect all of the other uses cases. Principled by this, the workshops mapped healthcare processes with criticalities emerged in the first step, refining the general list of requirements into functional and non-functional requirements to support the transmission of patient data through primary and secondary care, and through emergency and urgency care. *Kidney Chronic Disease* was discussed in light of referral and counter-referral procedures to address periodic follow-up. These were integrated with specific requirements for harmonization with the IT infrastructure in use, and refined with requirements for specialized care in under-served regions (e.g. periodic patient monitoring and treatment to attend chronic diseases and the corresponding referral and counter-referral flows through Primary, Secondary and Specialized care). The content analysis of video-recorded workshops are available in digital repository as supplemented material (see: Data availability).

A third step administered five online workshops to patient associations, aiming to validate the processes and, occasionally, to integrate perspectives on quality requirements (May to June 2024). The number of participants varied between 1 and 4 per workshop. Patient Associations are considered among the primary stakeholders since they typically involve both patients and family members of patients, and EHR in under-served areas “should have a high level of support by [..] community of users” and patients [26].

### Data analysis and application of the method

Data analyses from fieldwork involved a preliminary mapping of user journeys, utilizing a shared canvas using the Miro tool. Answers from the questionnaire respondents, in Portuguese, were translated into English by the native Portuguese researchers in the team. Occasionally automatic translation tools have been used as needed. This was followed by a content analysis applied to the answers that were common to all respondents, to identify common problems in all municipalities and specific subsets of problems for some municipalities.

A preliminary list of requirements was proposed for each use case. The map of processes explored in the workshop was translated into knowledge graphs, whose properties served to elicit a specific list of requirements compliant with FHIR and EU principles. A content analysis was performed on the transcriptions of workshop recordings, to capture the emphasis and engagement of participants on criticalities affecting patient handling, such as transmission of patient history, surgery scheduling and urgency request notifications in referral and counter-referrals. The rationale of requirements elicited from fieldwork was finally reported along with proposed evaluation metrics, following the “Volere Template” [57], and with putative priority ranking, according to the MoSCoW method.

## Results

Detailed findings from this study are available in the supplementary material section and a digital repository, which includes an analysis of common issues encountered across municipalities^7^. The following section provides a summary of the key insights.

### Main problems expressed

This study unveiled that the manual transmission of patient records remains a significant challenge in various municipalities, having an impact on all selected use cases. Below we list the most expressed problematic issues in the current health care system and associated journal management practices.

*Inadequate access to patient history* – Access to patient history remains typically isolated in the systems’ storage of the healthcare unit that attended the Patient. When referred to other units, Patients are required to repeat their symptoms to new HPCs, who may have no access to the relevant prior patient records from other health units or have very brief summaries from HCPs, carried along by Patients. This often results in incomplete patient records posing significant risks in managing emergency, urgency and specialized care cases. The administrative process to transfer data between units undergoes risks of data duplication of Patient records and maintenance costs (e.g. the practice can involve administrative staff bringing folders of papers from one unit to another).

*Refusal to review detailed anamnesis* – Some patient representatives reported a reluctance among doctors to review anamnesis presented on patients’ mobile devices or supporting documents, often citing time constraints. This issue motivates the development of protocols that issue a proof-of-reading certification by HCPs who accessed the anamnesis, aiming to enhance the accuracy of patient information carried through referrals (in agreement with the evaluation of Swedish EHR system in: [20]).

*Long waiting times and ineffective scheduling* – Patient handling is worsened by lack of resources and specialist staff in under-served municipalities, and by logistic problems to receive specialized care. For example, patients affected by chronic kidney diseases may have to travel 12 hours for dialysis treatment, due to the long distances involved and lack of equipped municipalities. Other factors impacting scheduling, reported by patient associations, include unclear communication to follow up with referrals and counter-referrals (e.g. lack of direction of which structure or specialist to contact; errors in notification of scheduled time of surgeries). In cases of missed appointments due to unavoidable delays, patients are de-prioritized in the queue lists, disregarding urgency, causing some patients to wait for months or even a whole year to receive specialized follow-up.

*Dismissed treatment due to travel time and costs* – While there is a home transport service for people requiring periodical follow-ups (e.g. dialysis for kidney failures), it needs to collect people from very distant locations. Patients reported the lack of significant financial support from public policy to cope with logistic issues (about 25 BRL [compensation], roughly equivalent to 5 USD); there is no policy response for addressing long-distance trips, such as those requiring flight or boat transportation. Some patients need to travel 300 kilometers to receive treatment. Lack of financial support and significant differences in logistical transportation increases the risk of Patients dropping therapy.

*Shortage of specialist care* – Patient representatives marked the lack of recruitment and retention of specialists in remote municipalities as a fundamental problem. The Patient associations motivated the need for a continuous learning campaign targeting the HCPs, rather than the patients, which was found in agreement with formative research and with Brazilian policies (see: Art. 3º, V in Portaria nº 389/GM/MS, de 13 de março de 2014 [5, 30]). For example, they mentioned that Patients affected by chronic kidney diseases know they should avoid salty alimentation regimes, but 5% of examined patients are already in urgent need of dialysis and the real problem is lack of specialists. They argue that the “preparation of general doctors is not adequate. They need to have more instructions [and formation to attend patients on site]”).

*Notification errors disrupting continuity of care* – Due to fragmented transmission of patient data in referrals and booking (e.g. municipalities are used to transfer patient data on paper at periodic intervals; booking of surgery is coordinated between administrative offices using spurious services, from email, notification and paper ), Patients associations and HCPs reported cases that did not receive confirmation of scheduled surgeries, thus being de-prioritized in the urgency list.

### Core requirements

For full results on the common problems found between municipalities (data not shown here), see the digital repository for supplementing material^8^.

Insights in this section are summarized for convenience in *Must-*, *Should-* and *Could-have* requirements. The first is considered non-negotiable for an embodiment of patient-centric and harmonized EHRs, legally compliant to secured electronic identification. The second is important to address the main problems affecting continuity of care, which emerged in this study. The latter may improve the experience of EHR functions, and should be enriched with quality requirements and capacity building targeting HCPs in follow up research, e.g., to address the pain-points of HCPs using EHR functionalities, evaluated in [20].

#### Must have

*Universal and secured access* – Any user interacting with the EHR must be uniquely and securely identified across the system. Typically, unique universal identifiers (UUID) are aligned with national security numbers - in the context of Brazil, national codes belonging to the “Cadastro de Pessoas Físicas” (CPF) or “Sistema Único de Saúde” (SUS) (e.g. see the requirements RU1.1 - RU1.6 in Appendix 1). Any item of the patient history must be linked to the UUIDs of the Patients, and carry the UUIDs of the authorized HCPs that referred the patient through services in primary, secondary and specialized care, in this way preventing data duplication and ensuring operational efficiency across different platforms (e.g. see SR40.2 - SR40.6, SR10.1 - SR10.3 in: Tables 2 and 3 in Appendix 1).

Integration UUID is fundamental to align data asynchronously in urgent situations such as emergencies: qualified HCPs staff must be able to update patient data post-admission; the system should alert in case of data duplication.

*Operational efficiency in referrals* – The system should allow healthcare providers to issue, update, and efficiently manage longitudinal patient records of referrals, biomarker tests and examination results. The expected impact is an improved workflow on behalf of the HCPs and a reduction of the waiting times on behalf of patients. Access to patient data must be granted only to authorized HCPs, and in aggregated form to IT for reporting tasks. The system must anyway be flexible to be configured to delegate permissions if required: as an example, under-served municipalities may have receptionists who can issue triage codes in place of nurses and nurses who can operate referrals in place of doctors.

*Robust referral patient management* – Patient-centric features (e.g. see: HPR1.1 - HPR1.2, SR41.1 in Tables 2 and 3 in Appendix 1) must enable comprehensive access to the patient history (e.g., PR55.1, HRP19) on behalf of both authorized HCPs and Patients; patient history must be accessible at any point of the patient journey, independently from location or type of received care. Patients must be capable of accessing their health data and the qualifications of healthcare providers who access their records; Patients must be capable of querying and viewing all necessary and complementary information related to follow-ups in referrals, such as contacts of doctors or of the units they are referred to or from, scheduling times and venues.

*Harmonization with current systems* – The data flow of patient records must interface with IT systems in use for data management to be compliant; in the case of Brazil, example are systems to handle booking of patients (e.g. “Sistema de Regulação” (SISREG) in Brazil); while not all systems may have an interface and some data be external to the EHR, the EHR should always trace the provenance of a patient handling procedure (marked by the corresponding UUID) - like a pointer to records that instead could be stored elsewhere.

The journal must interface with the prescription drugs dataset in use through the systems, and authorized HCPs must be capable of writing and reading necessary prescriptions, but never delete them: in case of errors, data must persist. In other words, the patient’s history cannot be amended retrospectively. Patient consent and awareness must also not impede user experience: for example, Patients requiring representatives (e.g. Patients who cannot read) or special assistance should never have interrupted care due to unauthorized permission towards their assistant.

We interpret it as receiving care is a priority over the security of own data, especially in an urgent context, and we anticipate that granting data consent may need to be scrutinized when granted to private companies, to ensure that a private operator may not discharge responsibilities to the Patients in case of data corruption (see also: auditing design principle in the EU eIDAS policy, prescribing that compliance of data security should be authorized, scrutinized and verifiable by third parties).

#### Should have

*Enhanced access, controls and permissions* – Municipalities should support access to geographic localization of certified HCPs (i.e. localize types of specialized professionals and where are authorized to operate), enhancing the trustworthiness and efficiency in referral of patients (e.g. finding detailed information about where and by whom patients are being treated). The system should allow access to Patient data also off-line or via their authorized device (e.g. via an eID-wallet).

*Advanced reporting capabilities* – Healthcare professionals should be able to report biomarker tests and medical evaluations directly in the patient’s EHR to consolidate medical data for accurate treatment and follow-up.

*Extension of referral types* – The architecture of the referral management should support modular extensions of data records. For example, referral objects should allow the creation of new fields describing the type of referrals (e.g. SR40.3), so to isolate referral types (e.g. referrals for triage; referrals to specialized care; referrals to external units). This should facilitate the digital transmission of referrals between internal and external units (SR41.1), reducing wait times and improving the overall patient referral process, as well as reconstructing the tree of attending Patients in complex cases and monitoring the effectiveness of care received.

*Automated notifications for appointments and criticalities* – HCPs should issue and receive notifications of referrals effortlessly (HCP13.3), ensuring that coordination is managed with minimal disruption. User experience, while not critical for a launch of MVPs system launch, significantly contributes to optimizing the operational efficiency and retention by practitioners.

*Agile management of data structures* – The data structure of the system should be modular: FHIR standards suggested to creation of templates describing instances of objects (like patients, HCPs, and prescriptions), which can be flexibly extended. An MVP may adopt FHIR-compliant APIs from cloud vendors (e.g. Amazon, Google), or implement its own APIs. In the latter case, an agile approach is expected to benefit from ontologies (e.g. via knowledge graphs) compliant with FHIR documentation, because they can flexibly align processes and data structures (e.g. from whom data is received), and facilitate maintenance. This study proposed a scaffolding for prototyping a knowledge graph of this kind.

#### Could have

*Indexing availability of qualified healthcare providers * – The capability for administrative or healthcare provider staff to view healthcare provider contact details directly in the system when on-boarding patients from received referrals (e.g. R96.3, HRP19) can support smoother transitions between care phases. This feature can improve the usability of EHR and decrease the likelihood of errors during patient transfers.

*Tracking of urgency solicitation requests* – In case of required hospitalization in external municipalities of higher complexity, practitioners are used to recur to emails and messaging applications from their smartphones to send periodic reminders of urgent requests for patient on-boarding or to communicate Patient transfer in an emergency. Integrating modules to track urgency solicitation requests sent via external channels (e.g. HCP13.3) or to automate the preparation of referrals and notifications to local and state regulation units (i.e. regional and statal “nucleo de regulacao”) (e.g. SR45) can support the practice of managing patient queues and prioritizing urgent cases, especially in high-demand scenarios.

*Tracking financial negotiations with private healthcare and logistic support* – The need for more significant financial support expressed by Patients’ representatives could be partly addressed by EHR modules for tracing financial negotiations between municipalities and private organizations, in such a way addressing requirements for monitoring the efficacy of health campaign [30, 35] (see: Requirements to address special care in remote rural areas, in Supportive Material): as example, partnerships with private clinics to address the lack of specialists and with private transportation services to reduce commuting time for specialized periodic care.

### Knowledge graphs

**Fig. 3:**
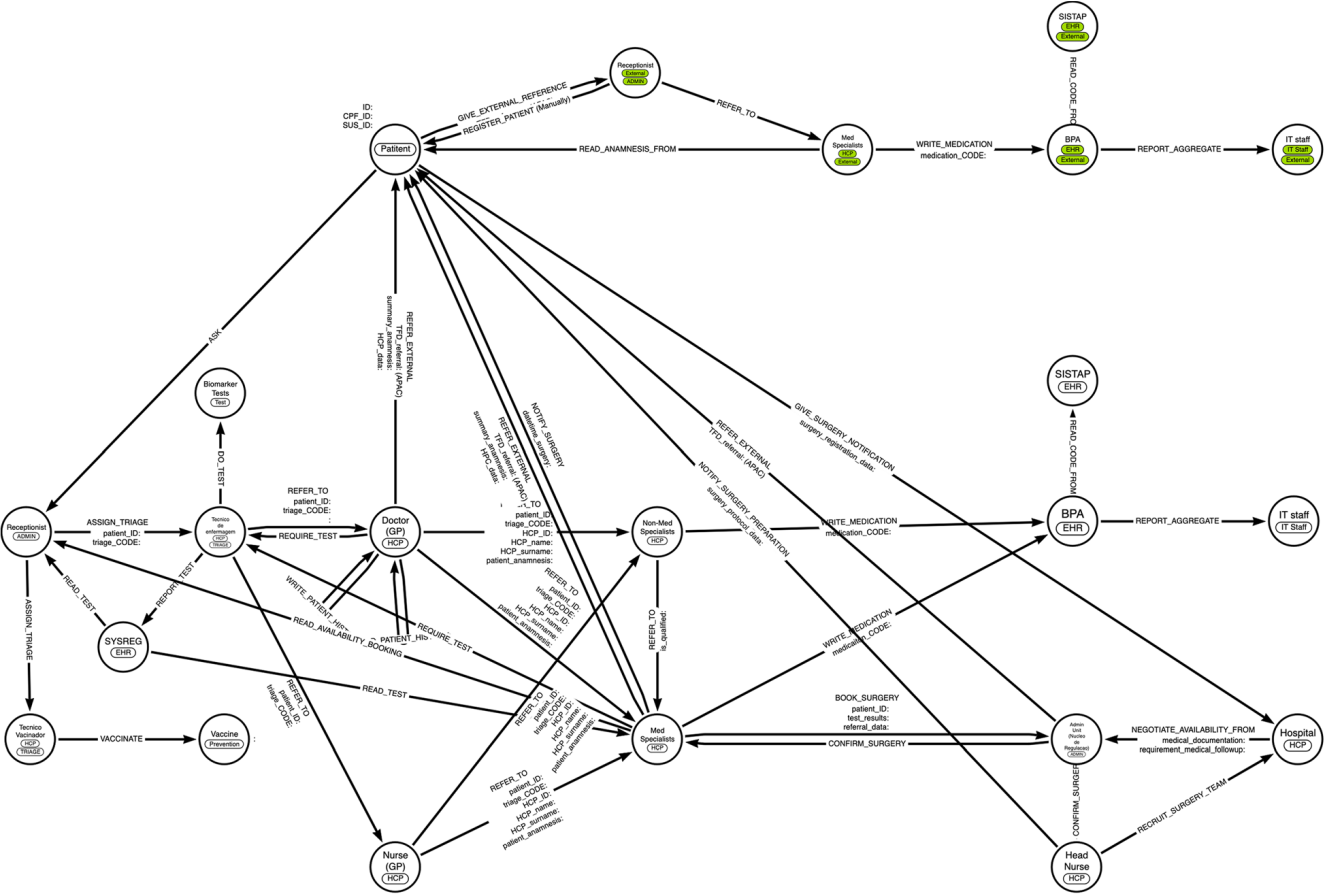
Knowledge Graphs Scheme for Referral and Counter-referrals. Scheme for Referral and Counter Referral Journal Module for primary and secondary care, extracted from fieldwork. Green labels represent an external SUS: the IT systems of SUS units are disconnected, and there is a lack of effective transmission of patient anamnesis between different structures. A query to bootstrap the model is available in supplementary material

**Fig. 4:**
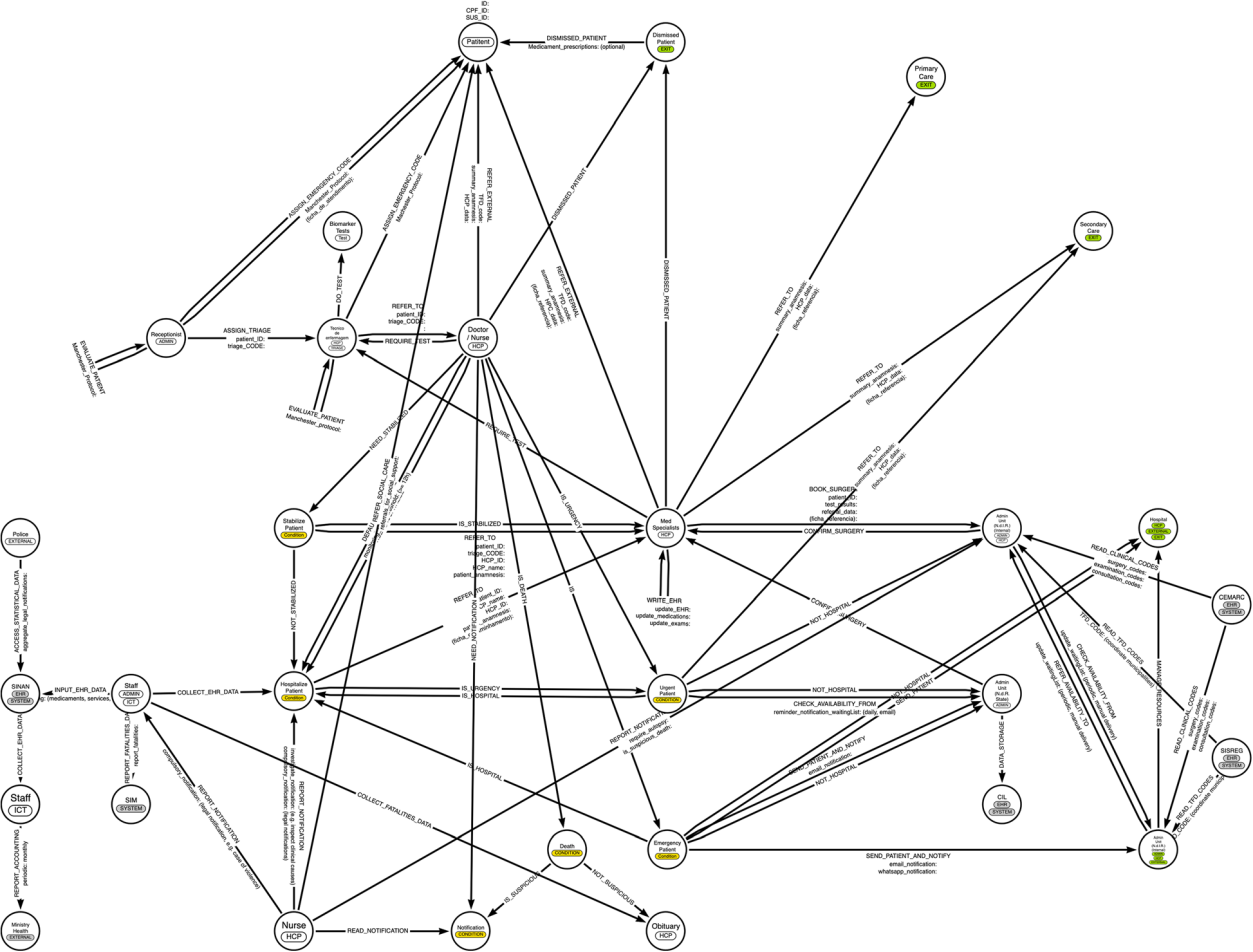
Knowledge Graphs Scheme supporting Emergency Cases (extending Referral and Counter-referrals). Scheme for Journal Module for Emergency cases, extracted from fieldwork. Hospitalization, Urgency and Emergency procedures rely on the efficiency of Referral and counter-referral systems. A query to bootstrap the model is available in supplementary material

Requirements were mapped into knowledge graphs (KG) for supporting patient-handling through referral and counter-referrals in primary and secondary care and in emergency cases; see, respectively: Figs. 3 and 4. Scripts to instantiate the knowledge graphs as journal module prototypes are provided in the repository of supplemental material, along with prescriptive requirements to structure the data schemes according to FHIR guidelines.

The design of KG followed the FHIR design principles to ease interoperability by retaining consistency with FHIR terminology and to ease maintenance by adopting a modular approach for data-structures and integrations (for details, see: Table “Design Principles - FHIR”, supplemental repository: Data availability). The networks of entities represents the healthcare objects and users and allow to combine semantic of medical and administrative procedures with the ontologies inheriting the medical taxonomy of FHIR standard [50]. This approach, that uses Web Ontology Language combined with description of logic (OWL-DL), is expected to facilitate versioning and backward compatibility with updates in medical ontologies [51], such as for managing multiple medication dispenses that needs to ensure decidability depending on prior history patients.

FHIR suggests to categorise resources into five compartments (i.e. logical grouping to access resources from servers) Patient, Encounter (i.e. information about the actual activities that occurred), Practitioner, RelatedPerson (i.e. attribution of information to users having personal or non-healthcare-specific professional relationship to a patient), and Device (see: [58] and, for granular functional and non-functional requirements, Table “Suggested Compartments Data Structures - FHIR” of supplemental repository Data availability). These KGs blueprinted patient-handling of primary and secondary health care and of emergency or urgency use cases and focused on Patient, Practitioner (i.e. HCPs, Admin staff) and Device (e.g. EHRs, IT Systems in use) compartment-types.

The nodes’ labels describe the entity types belonging to each compartment; the names describe the specific users (e.g. Generic Doctor or Medical Specialist, who are both labeled as “HCP” and belong to “Practitioner”). Encounter-type (e.g. Patients’ anamnesis and assigned triage codes) are encoded in the node’s properties, along with UUIDs of resources to track the “Provenance” of actions affecting the entities properties (for details, see: [59] and Table “Design Principles - FHIR” in the supplemental repository). The direction of the edges indicates who performs the action and who is the recipient of the action. This relationship is clarified through the labels on the edges. For example, the label “REFER_TO” on an edge between a nurse and a doctor means that the nurse is responsible for writing a referral entry in the journal system to document the referral of a patient, while the doctor is allowed to read that referral entry. Design requirements for data-structure query permissions and user type permissions are detailed in the Table “MVP Journal Requirements”; requirements for handling permissions via authenticated signatures compliant with FHIR are in the Tables “eID-Wallet - functional requirements”; “eID-Wallet - non-functional requirements” and “Handling digital signatures - FHIR” in the supplementary repository (see: Data availability).

Data structures of resources should be defined as extensible templates (see: “EHR System” and “Data Structure Requirements” sections in the Tables above). As example, in the Brazilian context there are different types of referrals, called *ficha(s)*, that are manually handed to the Patient when referring them to primary healthcare doctors, or specialistic care, or for other legal compliance. The digital twin of the paper-referrals should be designed as objects that inherit properties from a common template. The authorship tokens (i.e. UUIDs and other authorship descriptors, like name, surname and qualification of HPCs) must allow to reconstruct the patient journey at any given time, recalling authors of referrals, destiny of referrals, timestamp, patient-anamnesis and patient-history (e.g. prior anamnesis, medications and patient conditions such as allergies or prior surgeries).

Resources objects does not necessarily have to be stored in the KGs, but can be recalled via UUIDs identifiers and APIs interfaces. This is the typical case for supporting IT systems currently in use, whose name is reported for KGs’ nodes labeled as “EHR” or “System”. The system should anyhow persist the patient journey records in a way that is robust to failures of connectivity (e.g. through devices allowing off-line data-sharing if users are in proximity) and record formats should support data-sharing via XML or JSON formats, to support interoperability, and access to patient-documents and sharing should be tracebable (see section: “Sharing data privacy by design” in Table “eID Wallet - Functional Requirements”, and Table “Handling common use cases - FHIR” in supplementary material).

While logic could be programmed as functions of the system journal, we made explicit the logical conditions to handle patients also in the KGs (e.g. in case of urgency and emergency), along with the processes between patients and HPCs. This choice enabled us to validate the processes with representatives from healthcare providers (HCPs) and patient associations who did not have a technical background. This collaboration allowed for the co-creation of digitized patient referral and counter-referral processes that align with existing practices and can adapt to the diverse resources available among staff and IT systems across municipalities at local, regional, and state levels.

## Discussion

We argue that requirements for implementing referral and counter-referral modules are to be generalizable to under-served areas outside Brazil. The Brazilian strategy towards digital health care promotes actions to learn from other international experiences concerning Personal Health Record systems (see pp. 66 in [27]); accordingly, this study drew from the Swedish experience concerning ePrescription Journal adhering to FHIR standards and and best practices [18]; from the EU experience concerning eID enabling interoperability and transmission of EHR records; from Brazilian policies concerning the design of EHR modules addressing the provision of specialized care [29, 53, 54, 59] in emergent municipalities [30] , with special focus on kidney chronic diseases [31, 35, 60, 61] as an example where patients are mostly affected by manual transmission of patient history records and lack of followup from specialist care.

We maintain that the approach of combining KGs with fieldwork data facilitates the prototyping of MVP EHR systems in under-served areas characterized by manual transmission of patient data, overcoming some barriers in adopting electronic identification EHRs (limited to eID functionalities derived from the goals of patient-centricity and management of patient records) [37]. The knowledge graph can be executed to bootstrap a prototype (see: knowledge graph queries, in Supplement Material). Additionally, this study may facilitate the prototyping of EHR modules for handling specialized care, not with respect to the clinical procedures, but with respect to design integration with third-party services, under the requirements of patient-centricity and electronic identification (e.g. delegation of permissions to representatives, when specialized staff is not available, such as in the case of administrative staff executing triage procedures in understaffed health units).

Even if site selection to conduct the case study with municipalities from Maranhão constitutes a theoretical limitation for generalizing results towards other emergent areas, we considered our sample adequately large to draw sets of requirements satisfactory, not necessarily sufficient, for designing MVPs in emergent areas (e.g. municipalities operating in the context of lack of resources and remote areas), in particular aiding to limit the risks of contradictory needs depending on the resources that a municipality can count on (e.g. practices for handling patients can be different depending on the administrative types of municipalities, and our effort aimed to align these practices for continuity of care).

### Applicability of generic requirements from the EU to the Brazilian context

Among the EU requirements for eID authentication in digital health (eIDAS) (see Tables: “eID Wallet - Functional Requirements” and “eID Wallet - Non-Functional Requirements” in the supplementary repository, and [6]), we found interoperability and cross-border usability are highly relevant to the Brazilian context. The lessons from the EU’s diverse member states are valuable for Brazil, where healthcare services span vast, socio-economically varied regions. The focus on accessibility and interface simplicity aligns well with Brazil’s efforts to make healthcare more inclusive and user-friendly. These features can improve patient access to records, especially in under-served areas, and support informed decision-making through comprehensive views of health histories.

However, implementation-specific requirements for eID wallets are less directly applicable, as they must consider Brazil’s unique IT infrastructure. While data protection and security are critical - especially under data-protection regulations like the EU’s GDPR and Brazil’s LGPD - these should not come at the expense of user experience. In the Brazilian context, patient-centricity would benefit from eID wallet that support offline functionality and flexible permission allocation to adapt to staff shortages and resource constraints, common in smaller municipalities. For example, admin staff or nurses often perform functions typically handled by doctors. It is essential that patients and caregivers can easily access records, even if they lack literacy, connectivity, or smartphones. Valuable applicability could be found, beyond digital options as smartphone applications, in devices that work offline, are simple to use and allows physical identification (examples may be eID-cards or NFC technology). Initially, these solutions might coexist with manual paper-based systems, and healthcare and admin staff should find them intuitive. Insights on usability and expected benefits can be drawn from evaluations of Sweden’s eJournal systems [20].

Integrating knowledge graphs adds a valuable layer for managing infrastructure heterogeneity and identifying data-sharing, permissions, and interface requirements based on user practices. This enables granular, flexible data models for handling patients; referral and counter-referral paths could be stored locally and shared offline when proximity-based sharing is needed, if cloud authentication is unavailable (see “Attestation Management” and “Sharing of Data - Privacy by design” in Table “eID Wallet - Functional Requirements”, supplementary repository).

### Prioritizing digital harmonization of referral and counter-referrals

Specifically, the requirements for the use cases referral and counter-referral propose a layer to improve the efficiency for “access[ing] [EHR] management without regional access restrictions”, and enable integration of “multiple organizations [..] for scheduling appointments and exams” and the “knowledge extraction for improving diagnosis and evaluation of therapies” (see p. 77 in [27]), such as retrieval of anamnesis to prescribe therapies compliant with the patient history. This requirement addresses the strategic action “to enable the [Brazilian] National Healthcare Data Network (RDNS) to offer essential Digital Health services in the Country”, by harmonizing the design of EHRs with functional requirements to operate with SUS systems. An example is enabling transmission of EHR anamnesis through Primary, Secondary, and Specialist care (Referral and Counter-referral cases) to address the problem of ineffective or inefficient access to Patient health records.

The requirements for referral and counter-referral were focused on primary and secondary health care patient handling, in agreement with Brazilian policy that aims to ensure continuity of the citizen’s care when accessing health information, by starting harmonization with primary health care systems (“Conecte SUS Program”, GM/MS Ordinance no. 1.434, dated May 28, 2020; “Informatiza APS Program”, art. 504-A of Consolidation Ordinance no. 5/GM/MS, dated September 28, 2017; “Pilot Project Supporting the Informatization of Primary Healthcare”, Ordinance no. 2.984, dated November 11, 2019) (see: pp. 29; 32 in [28]).

Requirements and schemes for referral and counter-referrals enable a system continuity of healthcare through different units and types of specialization; by implication, we argue that our study supports a “portfolio [of offered services] (or considered necessary) to the population”, which is the first component to address Universal Health Care (UHC) highlighted by WHO. By implication, it can potentially contribute to increasing “the share of the population who can access [such services]” (e.g. population living in under-served or remote areas) and to lower “the share of direct costs that patients are required to pay to benefit from [such services]” (i.e. in case of traveling to transmit patient data) [1]. However, we anticipate that while we consider these requirements necessary, we do not consider them sufficient. We further anticipate the need for qualitative requirements and capacity building during the implementation and deployment of MVPs in digital health, which are out of the scope of this study.

### Limitations

A key consideration of digital transformation in healthcare is to assess if the digital technology supports the current processes, or if it requires a fundamental change instead. In the fieldwork of this study, participants were engaged to map problems and requirements focused on patient centricity; elicitation focused on management processes and harmonization of current technology. It is expected that our contribution mitigates the risks of disrupting organizational changes due to the introduction of EHRs adopting functionalities based on electronic identification (for example, unauthorized access to functionalities an HCP has been familiar with), however qualitative requirements addressing the user experience of such digital solutions, and capacity building in assisting their uptake, should be thoroughly covered in future implementations.

## Conclusion

In this paper we have systematically identified sets of critical requirements for a minimum viable product of an EHR system principled by patient-centricity, enabling digital transmission of and access to patient records through referral and counter-referral processes. These processes are deemed to lie at the core of the uninterrupted information flows in the care service, enabling retrieval and forwarding of relevant health history of patients whenever they seek medical attention.

Our results facilitate the generalization of essential requirements for digital referral and counter-referral processes applicable to emergent areas beyond the case described here. This adaptation involves implementing FHIR-compliant services and design principles that harmonize system integration for patient-centricity, incorporating APIs from local information systems.

Insights from the co-creation approach support that harmonization towards legacy system is strongly advised and discourage the introduction of systems designed from scratch, and a modular approach can assist the design of systems to gradually increase support of complex healthcare procedures.

Additionally, the study outlined necessary functionalities aimed at securing electronic identification, similar to eIDAS compliance, that enhance patient-centric operations, reducing innovation barriers related to the digitization of health. It also included processes to support continuity of care, mapped in the form of knowledge graphs that can be utilized to bootstrap the prototyping of such MVPs, with requirements guiding the implementation of FHIR-compliant data structures and APIs, addressing harmonization challenges. For an extensive set of requirements and a detailed description of each item, we invite readers to access the digital version of this study. Our findings provide insights into replicating MVP journals that can serve diverse operational and cultural contexts, focusing on common issues affecting patient-centricity in emerging areas.

## Data Availability

The datasets generated and/or analyzed during the current study are available in the repository ‘eSHARE’: https://su.drive.sunet.se/index.php/s/ dHEsN8p7zjRsg7o – All data generated or analyzed during this study are included in this published article and its supplementary information files on the digital repository. – The data generated includes analyses of questionnaires and requirements collected through workshops, content analysis of video-recorded material, code availability to instantiate the knowledge graphs. Collected primary data from questionnaires are anonymized. Videos recorded in workshops is not available to preserve anonymity. – The supplementary files includes: 1. sets of requirements to implement: minimum viable journal supporting selected use cases in primary, secondary and emergency care compliant with eIDAS electronic identification; 2. general requirements for healthcare continuity and prevention in remote areas (Kidney Chronic Diseases as special care case); 3. Knowledge Graph schemes for referral and counter- referral, and for emergency care use cases; 4. Analysis of problems emerged from questionnaires and workshops; 5. Questionnaire administered to HCPs. – Scripts to instantiate the knowledge graphs (Cypher syntax) are available in the repository.

## Appendix 1 Selected sets of requirements

Tables 2 and 3 presents, respectively, sets of requirements for supporting referral and counter-referral cases with transmission of patient history, and for handling patients in emergency cases. Each requirement includes a description (**D**), the rationale behind its purpose (**R**), and the fit criterion used for evaluation (**F**), following the “Volere Template”. If the **R** or **F** fields are missing, they inherit the rationale or fit criterion from the nearest preceding requirement that includes them. For full details, including MoSCoW evaluation, complete sets of requirements and their IDs categorization, see the digital version at: https://su.drive.sunet.se/index.php/s/dHEsN8p7zjRsg7o

**Table 2:** Requirements for exchanging patient history through Referral and Counter Referral cases

ID#	Requirement
*eID support*	
RU 1.1	**D:** Any User shall securely access by utilizing a unique universal identifier.
	**R:** To ensure each patient is uniquely identified and traceable throughout their care.
	**F:** The system shall be tested to avoid duplicate cases also in the condition of homonym and ensure anonymity of patients.
RU 1.2	**D:** HCP identifier shall include name, surname, qualification of the professional.
RU 1.3	**D:** HCP identifier shall support access to geographical localization (i.e., municipalities and healthcare centers where they operate).
RU 1.4	**D:** HCP identifier shall support access to the certified qualification of the Professional (i.e., municipalities and healthcare centers where they are authorized to operate).
RU 1.5	**D:** HCP identifier shall support the integration of personal contact details OR of the unit.
PR 55.1	**D:** Patient can access information about HCP qualification AND HCP contact details or HCP unit details associated with referrals, counter-referrals, and any decisions taken on their patient history.
R 96.3	**D:** Admin staff OR HCP staff shall be able to view HCP contact details, or Unit contact details in their place when on-boarding patients from received referrals.
HRP 19	**D:** HCP staff shall be able to view HCP contact details, or Unit contact details in their place when on-boarding Patients from received referrals if admin staff is not available.
SR 12.1	**D:** Ensure that data duplication is prevented by using secure login credentials.
RU 1.6	**D:** The patient’s Universal Identifier shall integrate and align with the CPF or SUS codes.
	**R:** To ensure that CPF and SUS codes can be aligned asynchronously, in cases of urgency, emergency, or death.
	**F:** The system shall be tested to allow updates of CPF, SUS, and patient data after their onboard in the ER, with credentials provided by Patients’ representatives or HCP staff.
*Triage*	
R96	**D:** Enable triage technicians to assign triage codes and report them directly to the EHR system.
	**R:** To standardize patient assessment and streamline the care pathway.
	**F:** Admin or HCP operator shall be able to onboard OR add a new entry to the Patient history in the EHR system.
SR 12.1	**D:** Enable HCP staff to report biomarker tests and medical evaluations on the Patient’s EHR.
	**R:** To consolidate patient medical data for accurate treatment and follow-up.
	**F:** The system shall be tested to record and retrieve biomarker results in the history of the Patient.
*Referrals*	
HPR 1.1	**D:** HCP professionals should be able to retrieve the Patient’s history at any point.
	**R:** To facilitate accurate referrals across various healthcare providers.
	**F:** Retrieval should be tested against Patient ID, CPF, and SUS, and all retrieved referrals should be linked to the correct patient records.
HPR 1.2	**D:** HCP professionals should be able to read consultation types and criticalities (e.g., previous or current medicaments and illnesses) that occurred in the Patient’s history.
	**R:** To facilitate monitoring in attending to the Patient.
	**F:** The system shall be tested by retrieving the history of received attendance of the Patients.
SR 40.2	**D:** Inherit all referral types (i.e., “fichas”) from a common referral template class compliant with FHIR documentation (e.g., attending, referral, external referral records - “ficha de atendimento”, “encaminhamento”, “referencia” intra- or extra-municipality).
	**R:** To allow reconstruction of the reference trees for attending the Patient, at local, regional, and state levels, following FHIR practices.
	**F:** The system shall be tested by retrieving the history of received attendance of the Patients.
SR 40.3	**D:** Support extension of referral types with fields functional to record the decision and its support (e.g., decision of hospitalization and anamnesis of the patient).
	**R:** To facilitate entry and retrieval of types of decisions over the clinical conditions of the Patient, at any specified datetime.
	**F:** Interfaces for issuing referrals shall be certified as complying with the paper referrals in use (e.g., “atendimento”, “encaminhamento”, “referencia”, and “TFD” for external attending, “notificacao”, “investigacao”).
SR 41.1	**D:** Enable digital transmission of referrals between internal and external units.
	**R:** To efficiently transmit referral requests between internal and external units.
	**F:** The system shall increase efficiency with respect to existing practices and shall diminish waiting time for issuing referrals.
SR 13.1	**D:** Ensure that the history of the Patient is collated under any condition even if records were acquired in different municipalities.
SR 45	**D:** Automate preparation of referrals and notifications to local and state regulation units (i.e., regional and state “nucleo de regulacao”).
	**R:** To ensure timely communication and coordination with regulatory bodies.
	**F:** The system shall increase efficiency with respect to the current transfer of referrals on paper.
HCP 13.3	**D:** Integrate a module to track urgency solicitation requests, sent via external channels.
	**R:** To trace solicitation requests for patients in the waiting lists, sent via external channels (e.g., email and WhatsApp).
	**F:** The system should increase efficiency in solving solicitation cases with respect to current practice.
SR 10.1	**D:** Ensure interoperability with SISREG systems for clinical attendance codes and municipality coordination.
	**R:** To integrate with existing regulation systems for efficient patient management.
	**F:** The system shall interface or be compliant with SISREG to coordinate with municipality codes.
SR 10.2	**D:** Ensure interoperability with SISTAP systems for clinical attendance codes and municipality coordination.
	**R:** To integrate with existing regulation systems for efficient patient management.
	**F:** The system shall interface or be compliant with SISTAP to manage procedures and medication records.
SR 40.6	**D:** Ensure interoperability with BPA systems for administrative data linkage.
	**R:** To integrate with existing regulation systems for efficient patient management.
	**F:** The system shall interface or be compliant with BPA to manage reporting and claims for funding.
HPR 2.3	**D:** HCP staff to issue and receive notifications of referrals effortlessly.
	**R:** To ensure smooth patient transfers and acceptance by external facilities.
	**F:** The system shall test that transfer requests and acceptance include all necessary clinical data AND the capacity of operators to uptake communication.
T1	**D:** Provide adequate training and system access to all involved actors, ensuring role-based access control.
	**R:** To ensure all users can effectively use the system and access only relevant information.
	**F:** Users shall pass a capacity-building test to operate in the system.
SR 24.1	**D:** Enable the creation of referrals and access to Patient records depending on the qualification of HCP staff.
	**R:** To ensure all users can securely use the system and access only relevant information.
	**F:** The system shall test that users can access required functionalities depending on their roles and permissions.
SR 39.1	**D:** Ensure all exchanged data (identifiers, emergency codes, test results, etc.) are standardized, secure, and comply with Brazilian privacy regulations.
	**R:** To protect patient data and comply with privacy laws.
	**F:** Data handling shall be tested against security standards and privacy laws.
SR 41.2	**D:** Facilitate on-boarding and retrieval of medical history with referrals (ficha da referencia) for patients needing care at other facilities (e.g., TFD, APAC).
SR 10.3	**D:** Enable integration with current systems for data storage of resources employed (e.g., CIL).
	**R:** To leverage existing infrastructure for better resource management, improving data accessibility, and reducing redundancy.
	**F:** The system shall be tested to integrate with CIL, demonstrating reduced redundancy maintenance.
R 96.1.4	**D:** Receptionists and Admin staff shall be granted permissions to issue triage codes and referral for on-boarding and routing the Patient (e.g., “ficha de atendimento”).
	**R:** To facilitate the initial patient assessment and routing in the ER, if required by a Unit.
	**F:** Usability tests to ensure receptionists and admin staff can issue and manage triage codes and referrals, within a desired error rate.
HPR 19.1	**D:** Nurses shall be granted permission to create and update triage codes, and referrals for routing the Patients for evaluations (e.g., “ficha de referencia”).
	**R:** To empower nursing staff to manage patient flow efficiently, adapting to dynamic clinical assessments.
	**F:** The system shall allow nurses to update and create triage codes and referrals accurately, with changes logged and traceable.
HPR 11.1	**D:** Nurses, doctors, and specialists shall be granted permission to create and update the patient’s EHR records (e.g., anamnesis) to prescribe biomarker tests.
	**R:** To ensure that medical professionals have the ability to document comprehensive medical histories and order necessary tests, enhancing diagnostic accuracy.
	**F:** Functional tests to verify that EHR updates and biomarker test prescriptions by authorized staff are correctly recorded and cannot be deleted.
HPR 11.2	**D:** Doctors and specialists shall be granted permission to create and update the Patient’s EHR records to prescribe medicaments (e.g., medicaments).
	**R:** To enable HCP staff to provide timely medication management and maintain accurate health records, improving treatment outcomes.
	**F:** Test cases to confirm that doctors and specialists can update EHR records and prescribe medications with full traceability.
HPR 13.1	**D:** Nurses, doctors, and specialists shall be granted permission to create and update the referrals for routing the Patient for further evaluations (e.g., “ficha de referencia”).
	**R:** To ensure that all relevant healthcare providers can contribute to and modify the patient’s care pathway as needed.
	**F:** Simulation test to ensure referrals for further evaluations are created, updated, and data integrity is maintained.
HPR 13.2	**D:** Doctors and specialists shall be granted permission to create requests for hospitalization AND flag it as urgent or emergent if needed.
	**R:** To allow for rapid and appropriate categorization of hospitalization needs, facilitating prioritized patient admissions.
	**F:** The system must allow the creation of hospitalization requests and correctly categorize them, with immediate updates to patient records.
HPR 7.1	**D:** Nurses, doctors, and specialists shall be granted permission to read all the referral types.
	**R:** To ensure comprehensive access to patient referral information, aiding in informed medical decision-making.
	**F:** Permissions matrix testing to ensure all authorized personnel can access and read referral types as per their roles.
HPR 12.1	**D:** Nurses shall be granted permission to create notification referrals (e.g., notifications to comply with legal requirements).
	**R:** To ensure compliance with legal and regulatory requirements through timely and proper notification of relevant health events.
	**F:** Compliance tests to verify that notification referrals meet legal standards and are correctly generated by nurses.
HPR 12.2	**D:** Doctors and Specialists shall be granted permission to revoke notification referral, but not delete its previous state.
	**R:** To maintain the integrity and traceability of medical records while allowing adjustments to current medical actions.
	**F:** Version control tests to ensure that revocation of notifications preserves the original record and is accessible only to authorized personnel.
HPR 12.3	**D:** Nurses, Doctors, and Specialists shall be granted permission to create investigation referrals (e.g., clinical investigation).
	**R:** To facilitate the initiation of necessary clinical investigations by qualified medical personnel, enhancing patient diagnosis and treatment.
	**F:** System functionality tests to confirm that investigation referrals can be created and are linked to the correct patient and clinical context.
R 95.1	**D:** Admin staff shall be able to read all notifications and EHR records for input and reporting on the current systems in use (e.g., SIM, SINAN).
	**R:** To enable administrative personnel to manage and report on health data effectively, supporting administrative tasks and compliance.
	**F:** Audit trails to verify that admin staff can access and report on EHR and notifications as needed without altering data integrity.
SAR 4.1	**D:** ICT staff shall be able to read, aggregate, and report notification and EHR records.
	**R:** To allow ICT staff to manage data effectively, ensuring accurate data reporting and system maintenance consistent with current IT requirements.
	**F:** The system shall be tested by operators to demonstrate reduced or at least equivalent maintenance.
SR 21.1	**D:** Patients’ EHR accessed by Admin and ICT staff should be anonymized.
	**R:** To protect patient confidentiality and comply with privacy regulations when non-medical staff access sensitive information.
	**F:** Security testing to ensure that any access to patient EHR by non-medical staff is anonymized, meeting privacy compliance.
*API Data Integrations (Brazil Specific)*	
DT 26	**D:** API for clinical records, interfacing with the SYSREG system.
	**R:** To preserve the integrity and continuity of medical records, ensuring historical data is maintained for ongoing patient care and analysis.
	**F:** Functional testing to confirm that the API effectively syncs with SYSREG systems.
DT 26.1	**D:** Enable read, write, and put functionalities for reading, editing, writing, and creating new entries.
	**R:** To facilitate real-time data exchange with the SYSREG system, improving data accuracy and patient care coordination.
DT 26.7	**D:** Update functionalities must not delete previous records (e.g., a clinical test for a biomarker cannot delete the previous test, only create a new instance and refer it to the same biomarker).
	**R:** To provide comprehensive interaction capabilities with EHR systems, allowing for flexible data management.
DT 27	**D:** API for medicaments accounting, harmonized with BPA and SISTAP systems.
	**R:** To preserve the integrity and continuity of medical records, ensuring historical data is aligned with medicaments and services utilized.
	**F:** Functional testing to confirm that the API effectively syncs with BPA and SISTAP systems.
DT 27.1	**D:** Enable read, write, and put functionalities for reading, editing, writing, creating, and deleting new entries (e.g., wrong entry of medication can be deleted).
	**R:** To integrate medication management with national systems in use, ensuring accuracy and compliance in drug dispensing and record-keeping.
DT 27.2	**D:** Enable aggregate functionalities for reporting and claiming of expenses for Admin and IT staff.
	**R:** To ensure data accuracy and flexibility in managing entries, allowing for corrections and updates as needed.
	**F:** Testing to ensure the system can aggregate and report financial data, supporting claims and expense management.
DT 27.3	**D:** Support traceability and retrieval of patient history, and retrieve prior associated tests and medications on operator request.
	**R:** To streamline financial processes and reporting within healthcare systems, facilitating easier management and reimbursement procedures.
	**F:** Testing to verify that all historical patient data, tests, and medications can be retrieved accurately by authorized personnel.
*Basic support for additional modules*	
SR 42	**D:** EHR should integrate modules to facilitate planning trips on behalf of Patients.
	**R:** To assist in the logistics of patient care, particularly for those requiring assistance with transportation to and from healthcare facilities.
SR 42.2	**D:** EHR should automate notification of new suggested appointments if urgent Patients miss appointments due to known systemic criticalities (e.g., transportation failures).
	**R:** To empower patients to communicate logistical challenges directly to healthcare providers, aiding in the scheduling and coordination of appointments.
SR 42.3	**D:** EHR should allow Patients to notify transportation issues to the healthcare unit.
	**R:** To ensure continuity of care by automatically rescheduling missed appointments due to unforeseen circumstances, aiming to reduce delays in urgent treatment.

**Table 3:** Requirements for supporting patient handling in emergency use cases

ID#	Requirement
*eID authorization*	
RU1.1	**D:** Any user shall securely access by utilizing a unique universal identifier.
	**R:** To ensure each patient is uniquely identified and traceable throughout their care.
	**F:** System shall be tested to avoid duplicate cases also in the condition of homonymy and anonymity of patients.
RU1.2	**D:** HCP identifier shall include name, surname, qualification of the professional.
RU1.3	**D:** HCP identifier shall support access to geographical localization (i.e., municipalities and healthcare centers where they operate).
RU1.4	**D:** HCP identifier shall support access to certified qualification of the Professional (i.e., municipalities and healthcare centres where they are authorized to operate).
RU1.5	**D:** HCP identifier shall support integration of personal contact details OR of the unit.
PR55.1	**D:** Patient can access information about HCP qualification AND HCP contact details or HCP unit details associated with referrals, counter-referrals, and any decisions taken on their Patient history.
R96.3	**D:** Admin staff OR HCP staff shall be able to view HCP contact details, or Unit contact details in their place, when onboarding patient from received referrals.
HRP19	**D:** HCP staff shall be able to view HCP contact details, or Unit contact details in their place, when on boarding patient from received referrals if admin staff is not available.
SR12.1	**D:** Ensure that data duplication is prevented by using secure login credentials.
RU1.6	**D:** Patient’s Universal Identifier shall integrate and align with the CPF or SUS codes.
	**R:** To ensure that CPF and SUS codes can be aligned asynchronously, in cases of urgency, emergency, or death.
	**F:** System shall be tested to allow the update of CPF, SUS, and patient data after their on-boarding in the ER, with credentials provided by Patients’ representatives or HCP staff.
RU1.2.1	**D:** Assign a temporary unique ID and allow for alignment with human supervision, if CPF or SUS are not available when Patient enters Emergency Units.
	**R:** To facilitate traceability of EHR records even when official identification documents (CPF or SUS) are unavailable.
*Triage*	
R96.1	**D:** Enable triage technicians to assign emergency codes based on the Manchester protocol and report emergency code directly to the EHR system.
	**R:** To prioritize patient care based on the severity of their condition.
	**F:** Emergency codes shall be tested with samplings of municipalities representing units of low, medium, and high complexity.
R96.1.1	**D:** Support the traceability of follow-ups of emergency codes depending on the complexity of the healthcare units.
	**R:** To monitor the effectiveness of ER referrals in the units.
	**F:** Emergency codes shall be tested with samplings of municipalities representing units of low, medium, and high complexity.
R96.1.2	**D:** Support triage evaluation and routing codes management (ficha de atendimento) according to the Manchester protocol.
	**R:** To standardize patient assessment and streamline the care pathway.
	**F:** The operator shall be able to read the triage code in the history of the referrals assigned to the Patient.
SR12.1	**D:** Enable HCP staff to report biomarker tests and medical evaluations on the Patient’s EHR.
	**R:** To consolidate patient medical data for accurate treatment and follow-up.
	**F:** System shall be tested to record and retrieve biomarker results in the history of the Patient.
*Referrals and Notifications*	
HPR1.1	**D:** HCP professionals should be able to retrieve the Patient’s history at any point.
	**R:** To facilitate accurate referrals across various healthcare providers.
	**F:** Retrieval should be tested against Patient ID, CPF, and SUS, and all retrieved referrals should be linked to the correct patient records.
HPR1.2	**D:** HCP professionals should be able to read consultation types and criticalities (e.g., previous or current medicaments and illnesses) that occurred in the Patient’s history.
	**R:** To facilitate critical care decisions and specialist referrals as needed.
	**F:** System shall be tested to retrieve decision outcomes and specialist consultations associated with Patient records (Patient History).
SR40.1	**D:** All referrals must track the HCP identifiers (universal Identifier, name, surname, qualification of the HCP staff); Patient Identifier; timestamp at the time of the decision; date and time at the time of future consultation if for primary and secondary care.
	**R:** To facilitate monitoring in attending the Patient.
	**F:** System shall be tested by retrieving the history of received attendance of the Patients.
SR40.2	**D:** Inherit all referral types (i.e., “fichas”) from a common referral template class compliant with FHIR documentation (e.g., attending, referral, external referral records - “ficha de atendimento”, “encaminhamento”, “referencia” intra- or extra-municipality).
	**R:** To allow the reconstruction of the reference trees for attending the patient, at local, regional, and state levels, following FHIR practices.
	**F:** System shall be tested by retrieving the history of received attendance of the patients.
SR40.3	**D:** Support the extension of referral types with fields functional to record the decision and its support (e.g., decision of hospitalization and anamnesis of the patient).
	**R:** To facilitate entry and retrieval of types of decisions over the clinical conditions of the patient, at any specified datetime.
	**F:** Interfaces for issuing referrals shall be certified as complying with the paper referrals in use (“fichas”). E.g., “atendimento”, “encaminhamento”, “referencia”, and “TFD” for external attending, “notificacao”, “investigacao”.
SR41.1	**D:** Enable digital transmission of referrals between internal and external units.
	**R:** To efficiently transmit referral requests between internal and external units.
	**F:** System shall increase efficiency with respect to existing practices (i.e., periodic manual delivery of external referrals (TFD) exchanged between regulation units (“nucleo de regulacao”) AND shall diminish waiting time for issuing referrals.
SR13.1	**D:** Ensure that the history of the patient is collated under any condition, even if records were acquired in different municipalities.
SR45	**D:** Automate preparation of referrals and notifications to local and state regulation units (i.e., regional and state “nucleo de regulacao”).
	**R:** To ensure timely communication and coordination with regulatory bodies.
	**F:** System shall increase efficiency with respect to the current transfer of referrals on paper.
SR45.1	**D:** Track the response of urgency solicitation requests sent via external channels.
	**R:** To trace solicitation requests for patients in the waiting lists, sent via external channels (e.g., email and WhatsApp).
	**F:** System should increase efficiency for solving solicitation cases with respect to current practice.
SR40.4	**D:** Inherit all notification referral types for clinical investigation (i.e., “fichas de investigacao”) from a common referral template class.
	**R:** To address standardizations of records according to FHIR practices.
	**F:** Data template and implementation shall be compatible with FHIR Classes or extend FHIR Classes.
SR40.5	**D:** Inherit all notification referral types for legal notification (i.e., “fichas de notificacao”) from a common referral template class, adopting FHIR.
SR46	**D:** Ensure Patient is uniquely identified upon entry to the ER even when official identification documents (CPF or SUS) are unavailable, and allows for later alignment and verification from authorized human supervision.
	**R:** To address standardizations of records according to FHIR practices.
	**F:** Data template and implementation shall be compatible with FHIR Classes or extend FHIR Classes.
SR10.1	**D:** Ensure interoperability with CEMARC systems for clinical attendance codes.
	**R:** To integrate with existing regulation systems for efficient patient management.
	**F:** System shall interface or be compliant with CEMARC to integrate surgery, examination, and consultancy codes and procedures.
SR10.2	**D:** Ensure interoperability with SISREG systems for clinical attendance codes and municipality coordination.
	**R:** To integrate with existing regulation systems for efficient patient management.
	**F:** System shall interface or be compliant with SISREG to coordinate with municipality codes.
HPR2.3	**D:** HCP staff should issue and receive notifications of referrals effortlessly.
	**R:** To ensure HCP staff effort to issue, receive, or be notified of a referral is not higher than current practices.
	**F:** Sampled HCP staff shall pass a test to be able to execute, receive, and operate a digitized referral within desired time thresholds.
HCP16.1	**D:** Support the registration and notification of special cases (deceased, mandatory notifications).
	**R:** To comply with legal requirements and ensure appropriate case handling.
	**F:** System must integrate with SINAN and SIM systems for death and mandatory case notifications.
T1	**D:** Provide adequate training and system access to all involved actors, ensuring role-based access control.
	**R:** To ensure all users can effectively use the system and access only relevant information.
	**F:** Users shall pass a capacity-building test to operate in the system.
SR24.1	**D:** Enable the creation of referrals and access to Patient records depending on the qualification of HCP staff.
	**R:** To ensure all users can securely use the system and access only relevant information.
	**F:** System shall test that users can access required functionalities depending on their role and permissions.
SR39.1	**D:** Ensure all exchanged data (identifiers, emergency codes, test results, etc.) are standardized, secure, and comply with Brazilian privacy regulations.
	**R:** To protect patient data and comply with privacy laws.
	**F:** Data handling shall be tested against security standards and privacy laws.
SR39.2	**D:** Enable integration with current systems for data aggregation and periodic reporting to the Health Ministry (i.e., SINAN, SIM).
	**R:** To provide accurate and timely health data reporting to the government.
	**F:** Referrals must be easily created, including all necessary historical and anamnesis data.
SR41.2	**D:** Facilitate on-boarding and retrieval of medical history with referrals (“ficha da referencia”) for patients needing care at other facilities (e.g., TFD, APAC).
	**R:** To facilitate continued care at the most appropriate facility.
	**F:** System shall be tested that referrals between external units allow reconstructing Patient History; retrieving anamnesis, medications, consultations done in other municipalities; retrieving the contact and allowing contact with the specialists who attended the Patient.
SR10.3	**D:** Enable integration with current systems for data storage of resources employed (e.g., CIL).
	**R:** To leverage existing infrastructure for better resource management, improving data accessibility, and reducing redundancy.
	**F:** System shall be tested to integrate with CIL, demonstrating reduced redundancy maintenance.
R96.1	**D:** Admin staff shall be granted permission to collate Patient ID with temporary ID of Patients unable to provide their credentials.
	**R:** To ensure continuous patient care and data accuracy when full identification is temporarily unavailable.
	**F:** Audit test to confirm that admin staff can collate and manage IDs without unauthorized access.
R96.2	**D:** Receptionists shall be granted permissions to issue triage codes.
	**R:** To facilitate the initial patient assessment and routing in the ER, if required by a Unit.
	**F:** Usability tests to ensure receptionists and admin staff can issue and manage triage codes and referrals, within a desired error rate.
HPR19.1	**D:** Nurse shall be granted permissions to create and update triage codes, and referrals for routing the Patients for evaluations (e.g., “ficha de referencia”).
	**R:** To empower nursing staff to manage patient flow efficiently, adapting to dynamic clinical assessments.
	**F:** System shall allow nurses to update and create triage codes and referrals accurately, with changes logged and traceable.
HPR11.1	**D:** Nurse, Doctors, and Specialists shall be granted permissions to create and update the Patient’s EHR records (e.g., anamnesis) to prescribe biomarker tests.
	**R:** To ensure that medical professionals have the ability to document comprehensive medical histories and order necessary tests, enhancing diagnostic accuracy.
	**F:** Functional tests to verify that EHR updates and biomarker test prescriptions by authorized staff are correctly recorded and cannot be deleted.
HPR11.2	**D:** Doctors and Specialists shall be granted permissions to create and update the Patient’s EHR records to prescribe medicaments (e.g., medicaments).
	**R:** To enable HCP staff to provide timely medication management and maintain accurate health records, improving treatment outcomes.
	**F:** Test cases to confirm that doctors and specialists can update EHR records and prescribe medications with full traceability.
HPR13.1	**D:** Nurse, Doctors, and Specialists shall be granted permissions to create and update the referrals for routing the Patient for further evaluations (e.g., “ficha de referencia”).
	**R:** To ensure that all relevant healthcare providers can contribute to and modify the patient’s care pathway as needed.
	**F:** Simulation test to ensure referrals for further evaluations are created, updated, and data integrity is maintained.
HPR13.2	**D:** Doctors and Specialists shall be granted permissions to create requests for hospitalization AND flag it as urgent or emergent if needed.
	**R:** To allow for rapid and appropriate categorization of hospitalization needs, facilitating prioritized patient admissions.
	**F:** System must allow the creation of hospitalization requests and correctly categorize them, with immediate updates to patient records.
HPR7.1	**D:** Nurse, Doctors, and Specialists shall be granted permissions to read all the referral types.
	**R:** To ensure comprehensive access to patient referral information, aiding in informed medical decision-making.
	**F:** Permissions matrix testing to ensure all authorized personnel can access and read referral types as per their roles.
HPR12.1	**D:** Nurse shall be granted permissions to create notification referrals (e.g., notifications to comply with legal requirements).
	**R:** To ensure compliance with legal and regulatory requirements through timely and proper notification of relevant health events.
	**F:** Compliance tests to verify that notification referrals meet legal standards and are correctly generated by nurses.
HPR12.2	**D:** Doctors and Specialists shall be granted permission to revoke notification referral, but not delete its previous state.
	**R:** To maintain the integrity and traceability of medical records while allowing adjustments to current medical actions.
	**F:** Version control tests to ensure that revocation of notifications preserves the original record and is accessible only to authorized personnel.
HPR12.3	**D:** Nurse, Doctors, and Specialists shall be granted permissions to create investigation referrals (e.g., clinical investigation).
	**R:** To facilitate the initiation of necessary clinical investigations by qualified medical personnel, enhancing patient diagnosis and treatment.
	**F:** System functionality tests to confirm that investigation referrals can be created and are linked to the correct patient and clinical context.
R95.1	**D:** Admin staff shall be able to read all notifications and EHR records for input and reporting on the current systems in use (e.g., SIM, SINAN).
	**R:** To enable administrative personnel to manage and report on health data effectively, supporting administrative tasks and compliance.
	**F:** Audit trails to verify that admin staff can access and report on EHR and notifications as needed without altering data integrity.
SAR4.1	**D:** ICT staff shall be able to read, aggregate, and report notification and EHR records.
	**R:** To allow ICT staff to manage data effectively, ensuring accurate data reporting and system maintenance consistent with current IT requirements.
	**F:** System shall be tested by operators to demonstrate reduced or at least equivalent maintenance.
SR21.1	**D:** Patients’ EHR accessed from Admin and ICT staff should be anonymized.
	**R:** To protect patient confidentiality and comply with privacy regulations when non-medical staff access sensitive information.
	**F:** Security testing to ensure that any access to patient EHR by non-medical staff is anonymized, meeting privacy compliance.
*API Data Integrations*	
DT1	**D:** API for clinical records, interfacing with SYSREG system.
	**R:** To preserve the integrity and continuity of medical records, ensuring historical data is maintained for ongoing patient care and analysis.
	**F:** Functional testing to confirm that the API effectively syncs with SYSREG systems.
DT1.1	**D:** Enable read, write, put functionalities for reading, editing, writing, and creating new entries.
	**R:** To facilitate real-time data exchange with the SYSREG system, improving data accuracy and patient care coordination.
DT1.2	**D:** Update functionalities must not delete previous records (e.g., a clinical test for a biomarker cannot delete the previous test, only create a new instance and refer it to the same biomarker).
	**R:** To provide comprehensive interaction capabilities with EHR systems, allowing for flexible data management.
DT2	**D:** API for medicaments accounting, harmonized with BPA and SISTAP systems.
	**R:** To preserve the integrity and continuity of medical records, ensuring historical data is aligned with medicaments and services utilized.
	**F:** Functional testing to confirm that the API effectively syncs with BPA and SISTAP systems.
DT2.1	**D:** Enable read, write, put functionalities for reading, editing, writing, creating, and deleting new entries (e.g., wrong entry of medication can be deleted).
	**R:** To integrate medication management with national systems in use, ensuring accuracy and compliance in drug dispensing and record-keeping.
DT2.2	**D:** Enable aggregate functionalities for reporting and claiming of expenses for Admin and IT staff.
	**R:** To ensure data accuracy and flexibility in managing entries, allowing for corrections and updates as needed.
	**F:** Testing to ensure the system can aggregate and report financial data, supporting claims and expense management.
DT2.3	**D:** Support traceability and retrieval of patient history, and retrieve prior associated tests and medications on the operator request.
	**R:** To streamline financial processes and reporting within healthcare systems, facilitating easier management and reimbursement procedures.
	**F:** Testing to verify that all historical patient data, tests, and medications can be retrieved accurately by authorized personnel.
*EHR Criticalities*	
SR14.1	**D:** Ensure that events are reported in the EHR of the patient IF Stabilized OR Hospitalized OR Dismissed OR referred to other units.
SR13.2	**D:** Ensure that criticality of Patient is updated in the EHR, IF Urgent OR Emergency.
SR42.1	**D:** Facilitate prioritization of patients in waiting lists by inheriting flags Urgent in all derivative functionalities used to handle appointments.

## Abbreviations

APIs: Application Programming Interfaces
APS: Atenção Primária á Saúde (Brazilian Primary Health Care)
CPF: Cadastro de Pessoas Físicas (Registry of Private Individuals)
eID: electronic identity
eIDAS: Electronic Identification And Trust Services
EHR: Electronic Health Records
EU: European Union
EUDI: European Digital Identity
FHIR: Fast Healthcare Interoperability Resources
GDPR: General Data Protection Regulation
GM/MS: Gabinete do Ministro / Ministério da Saúde (Office of the Minister / Ministry of health of Brazil)
HCPs: Healthcare Professionals
IT: Information Technology
KG: Knowledge Graph
LGPD: Lei Geral de Proteção de Dados
MVP: Minimum Viable Product
OWL-DL: Web Ontology Language - Description Logic
RDNS: Rede Nacional de Dados em Saúde (Brazilian Healthcare Data Network)
REST: Representational State Transfer
SISREG: Sistema de Regulação (Brazilian System to manage demand and offer of healthcare)
SUS: Sistema Único de Saúde (Brazilian public Unified Health System)
UHC: Universal Health Coverage
UUID: Unique Universal Identifiers
WHO: World Health Organization

## Footnotes

1 “Electronic Data Interchange for Administration, Commerce and Transport” is a syntax defined by the United Nations (UN) which defines “standards, directories, and guidelines for the electronic interchange of structured data, between independent computerized information systems”. See: https://unece.org/trade/uncefact/introducing-unedifact

2 GM/MS Ordinance no. 1.434, May 28, 2020

3 art. 504-A of Consolidation Ordinance no. 5/GM/MS, September 28, 2017

4 Ordinance no. 2.984, November 11, 2019

5 Distrito Federal, São Paulo, Rio Grande do Sul, Rio de Janeiro, Tocantins, Minas Gerais, Mato Grosso, Mato Grosso do Sul, and Piauí

6 https://miro.com/

7 https://su.drive.sunet.se/index.php/s/dHEsN8p7zjRsg7o

8 https://su.drive.sunet.se/index.php/s/dHEsN8p7zjRsg7o
